# Selective Extraction of ω-3 Fatty Acids from *Nannochloropsis* sp. Using Supercritical CO_2_ Extraction

**DOI:** 10.3390/molecules24132406

**Published:** 2019-06-29

**Authors:** Gian Paolo Leone, Roberto Balducchi, Sanjeet Mehariya, Maria Martino, Vincenzo Larocca, Giuseppe Di Sanzo, Angela Iovine, Patrizia Casella, Tiziana Marino, Despina Karatza, Simeone Chianese, Dino Musmarra, Antonio Molino

**Affiliations:** 1 ENEA, Italian National Agency for New Technologies, Energy and sustainable economic Development. Department of Sustainability–CR Casaccia. Via Anguillarese 301, 00123 Rome (RM), Italy; 2 ENEA, Italian National Agency for New Technologies, Energy and Sustainable Economic Development, Department of Sustainability-CR Trisaia, SS Jonica 106, km 419+500, 7026 Rotondella, Italy; 3 ENEA, Italian National Agency for New Technologies, Energy and Sustainable Economic Development, Department of Sustainability-CR Portici, P. Enrico Fermi, 1, 80055 Portici, Italy; 4 Department of Engineering, University of Campania “Luigi Vanvitelli”, Real Casa dell’Annunziata, Via Roma 29, 81031 Aversa, Italy

**Keywords:** microalgae, Supercritical CO_2_ extraction, lipid, fatty acids, eicosapentaenoic acid, docosahexaenoic acid, nutraceutical

## Abstract

In this article, microalgae *Nannochloropsis* sp. was used for fatty acid (FA) extraction, using a supercritical fluid-carbon dioxide (SF-CO_2_) extraction method. This study investigated the influence of different pre-treatment conditions by varying the grinding speed (200–600 rpm), pre-treatment time (2.5–10 min), and mixing ratio of diatomaceous earth (DE) and *Nannochloropsis* sp. biomass (0.5–2.0 DE/biomass) on FAs extraction. In addition, the effect of different operating conditions, such as pressure (100–550 bar), temperature (50–75 °C), and CO_2_ flow rate (7.24 and 14.48 g/min) on eicosapentaenoic acid (EPA) and docosahexaenoic acid (DHA) recovery, was analyzed. Experimental data evidenced that, keeping constant the extraction conditions, the pre-treatment step enhanced the FAs extraction yield up to 3.4 fold, thereby the maximum extracted amount of FAs (61.19 mg/g) was attained with the pre-treatment with a ratio of DE/biomass of 1 at 600 rpm for 5 min. Moreover, by increasing both SF-CO_2_ pressure and temperature, the selectivity towards EPA was enhanced, while intermediate pressure and lower pressure promoted DHA recovery. The highest amount of extracted EPA, i.e., 5.69 mg/g, corresponding to 15.59%, was obtained at 75 °C and 550 bar with a CO_2_ flow rate of 14.48 g/min, while the maximum amount of extracted DHA, i.e., ~0.12 mg/g, equal to 79.63%, was registered at 50 °C and 400 bar with a CO_2_ flow rate of 14.48 g/min. Moreover, the increased CO_2_ flow rate from 7.24 to 14.48 g/min enhanced both EPA and DHA recovery.

## 1. Introduction

Microalgae are unicellular photosynthetic microorganisms responsible for at least 32% of global photosynthesis, and nearly half a generation of the atmospheric oxygen [1]. Microalgae can be grown in controlled conditions, which could produce a higher amount of biomass with a desired biochemical composition and eliminating the risk of chemical contamination of the biomass. Therefore, microalgae research has gained significant importance in recent years [2]. Microalgae are rich in biomolecules, such as high-quality protein, essential amino acids, fatty acids (FAs), carotenoids and pigments with high nutraceutical value [3]. In particular, the production of FAs, including long chain polyunsaturated FAs [such as eicosapentaenoic acid (EPA) and docosahexaenoic acid (DHA)], has to be investigated in depth. EPA and DHA cannot be synthesized by the human body, while different organisms, mainly from aquatic origin, provide to their synthesis. Therefore, EPA and DHA are considered as essential dietary nutrients, needed for the correct human metabolism. Both molecules provide health benefits, such as the reduction of cardiovascular diseases, including arrhythmia, stroke, and high blood pressure, and can ameliorate renal diseases, depression, dementia, rheumatoid arthritis, and asthma. Moreover, these molecules are essential for the normal fetal brain development, as well as growth and development of infants/children [2,4,5].

For lipids production, microalgae are considered potential sources, since they contain a higher amount of EPA and DHA. Among the known microalgae species, *Nannochloropsis* (eustigmatophyte) species are considered the most promising EPA and DHA natural producers [1,6]. However, the extraction of EPA and DHA with high purity is still challenging, due to the concomitant extraction of other lipids, which limit their direct use in the nutraceutical industry. Among the extraction techniques, supercritical fluid-carbon dioxide (SF-CO_2_) extraction method is considered an efficient one to achieve high extraction together with the maximum purity. Additionally, SF-CO_2_ extraction majored as green extraction approach, due to less use of organic solvents, since it adopts low temperature and high pressure [7,8,9,10]. In addition, CO_2_ in supercritical conditions has low viscosity, low surface tension, high diffusivity, and good density and it is also non-toxic, non-flammable, cheap, widely available, chemically inert under several conditions, and gaseous at normal pressure and temperature, eliminating the step of solvent evaporation after extraction [11]. The basic principle of SF-CO_2_ is achieving a supercritical phase that is beyond the critical point of a fluid, in which the meniscus (that separates the liquid and vapor phases) disappears, and leaves a single homogeneous phase [12,13,14]. Moreover, the changes of the thermophysical properties transform the fluid into a super-solvent and thus, could improve extraction efficiency [15].

Extraction temperature and pressure play a significant role in the solubility of solutes in the solvent, which mainly depends on the chemical properties of extractive target compounds. For example, astaxanthin and lutein show thermal degradation at high temperature, therefore working conditions need to be optimized for an optimal extraction [16,17,18,19]. The flow rate of CO_2_ defines the residence time for the contact between the solute and the solvent, which have various effects on the selectivity of bioactive compounds and extraction efficiency. To the best of authors’ knowledge, only a few reports in the literature on the role of CO_2_ flow rate on the selectivity of bioactive compounds and extraction efficiency exist [16,17,20,21,22]. Machmudah et al. [22] investigated the effect of CO_2_ flow rate (2–4 mL/min) at 50 MPa and 50 °C for astaxanthin recovery from *H. pluvialis*, and they found that the amount of total extract could be increased, whereas the amount of astaxanthin in the extract almost remained unchanged. Ruen-Ngam et al. [21] investigated the role of CO_2_ flow rate on lutein extraction at 40 °C and 40 MPa and reported that extraction of lutein was improved by increasing CO_2_ flow rate from 2 mL/min up to an optimum level and then tended to decrease in extraction efficiency at greater CO_2_ flow rate. 

In this study, the *Nannochloropsis* sp. biomass was used for the extraction of EPA and DHA through SF-CO_2_ technology after mechanical cell disruption. The purpose of this study was to evaluate the effect of different operating conditions, such as pressure (100–550 bar), temperature (50–75 °C), and CO_2_ flow rate (7.24 and 14.48 g/min) on the EPA and DHA recovery. Prior to SF-CO_2_ extraction, the biomass was pre-treated and mechanical cell disruption conditions were optimized. 

## 2. Results

### 2.1. Effect of Mechanical Pre-Treatment on Fatty Acid Recovery 

**Figure 1:**
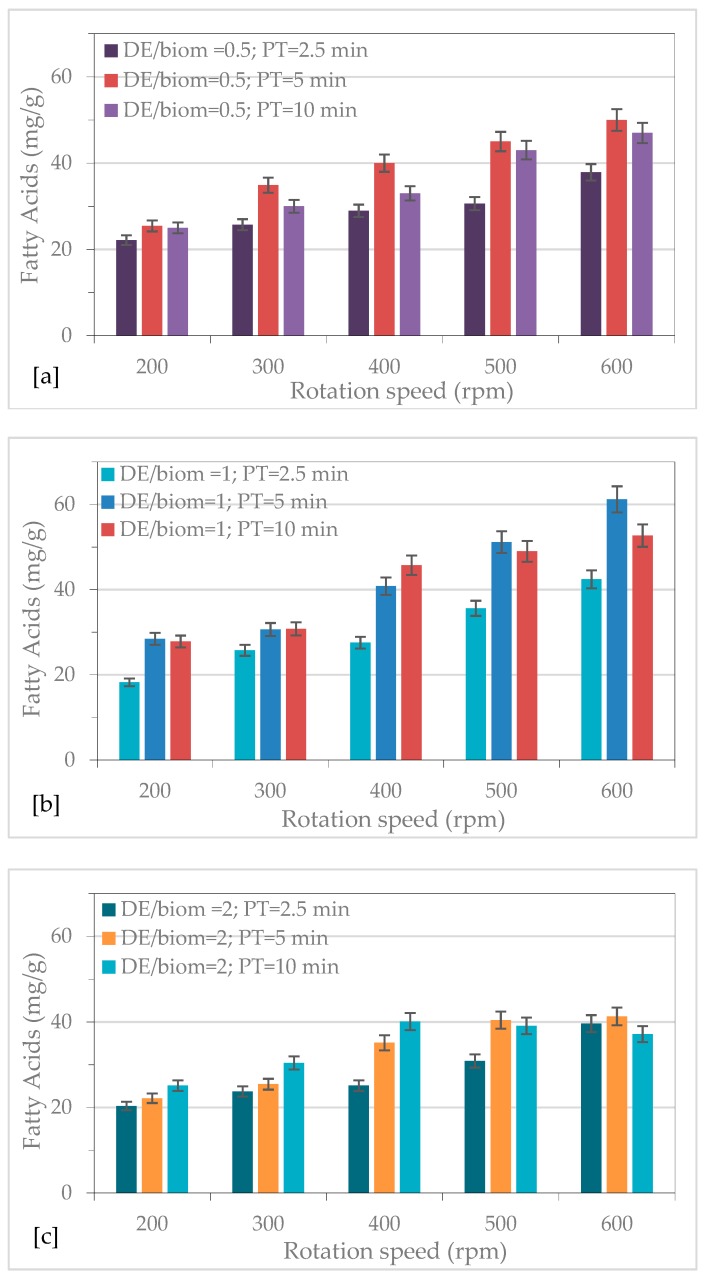
Effect of rotation speed and pre-treatment time on fatty acids (FAs) recovery with diatomaceous earth (DE)/biomass mixing ratio of: (**a**) 0.5; (**b**) 1; (**c**) 2.

Mechanical pre-treatment using ball milling was considered as an efficient method to enhance the extraction yield of intracellular compounds from microalgae biomass [13,23,24,25]. Figure 1 shows the effect of pre-treatment conditions on FAs recovery from *Nannochloropsis* sp. The obtained results showed that the extraction yield of FAs gradually increased with the increase of grinding speed. The lower extraction yield of FAs was obtained at 1.0 DE/biomass mixing ratio and 200 rpm for 2.5 min, while the 1.0 DE/biomass mixing ratio led to the maximum FAs recovery by performing the pre-treatment at 600 rpm for 5 min. Cheng et al. [26] found that the bead-beating disrupted *Pavlova* sp. biomass enhanced 3.0 fold FA methyl ester extraction yield during SF-CO_2_ extraction.

### 2.2. Effect of Extraction Condition on Extraction Yield and Lipids Recovery

Extraction temperature and pressure play a crucial role in the economy of the process, as they heavily affect the recovery of intracellular compounds from different matrixes. Temperature and pressure affect the supercritical CO_2_ process in a complex way, due to their combined effect on solvent density and solute vapor pressure. In fact, the solute vapor pressure tends to increase with temperature raising the solubility, while temperature displays an opposite effect on the solvent density and solubility. The influence of temperature and pressure of SF-CO_2_ extraction, with a CO_2_ flow rate of 7.24 and 14.48 g/min on the recovery of fatty acids, was studied. Table 1 shows the effect of extraction conditions of extraction yield and lipid recovery. The maximum extraction yield of 94.28 mg/g, with total lipid extract equal to 18.39 mg/g, was obtained at 75 °C and 550 bar with a CO_2_ flow rate of 14.48 g/min. The maximum amount of SFAs, MUFAs and PUFAs were also attained during SF-CO_2_ extraction at 75 °C and 550 bar with a CO_2_ flow rate of 14.48 g/min. In terms of purity, the lower the pressure, the higher the purity, as the highest lipid purity was found at 75 °C and 100 bar with a CO_2_ flow rate of 14.48 g/min. Literature reports that lipid recovery is enhanced by temperature and pressure increases [27,28]. The quantitative analysis of FAs highlighted how SF-CO_2_ extraction conditions strongly affected the recovery of SFAs, MUFAs and PUFAs. Extraction pressure showed a significant influence on the recovery of a different class of FAs; increasing pressure from 100 to 550 bar, extraction of FAs increased. Results in Table 1 show that FAs recovery increases by increasing temperature and pressure. Among the tested CO_2_ flow rates, 14.48 g/min enhanced the extraction yield of FAs for all the operative pressure and temperature investigated. 

For SF-CO_2_ extraction, 100 bar is the so-called cross-over pressure, which suggests that at higher pressures, the recovery is enhanced, due to the increase of vapor pressure, instead of the density. Such a phenomenon, supported by several experimental studies, is related to the fact that at lower pressures, the expected increase in oil fugacity (with the increase of temperature) is overcome by the decrease in SF-CO_2_ density and, therefore, by the decrease of its solvent power [29,30]. Determination of crossover pressure appears interesting to better understand supercritical fluid phase phenomena in a region where the solubility is extremely sensitive to pressure [31]. 

It is worth highlighting that in this work, the attention was devoted to EPA, DHA and lipids extraction and characterization. However, it might be possible to estimate that the other algal components, such as carbohydrates (mainly cellulose and lignin) are not solubilized by SF-CO_2_, while the soluble fraction of proteins and fibers could represent a part of the remaining extract.

### 2.3. Effect of Different Pressure and Temperature on EPA Recovery with a CO_2_ Flow Rate of 7.24 and 14.48 g/min

**Figure 2:**
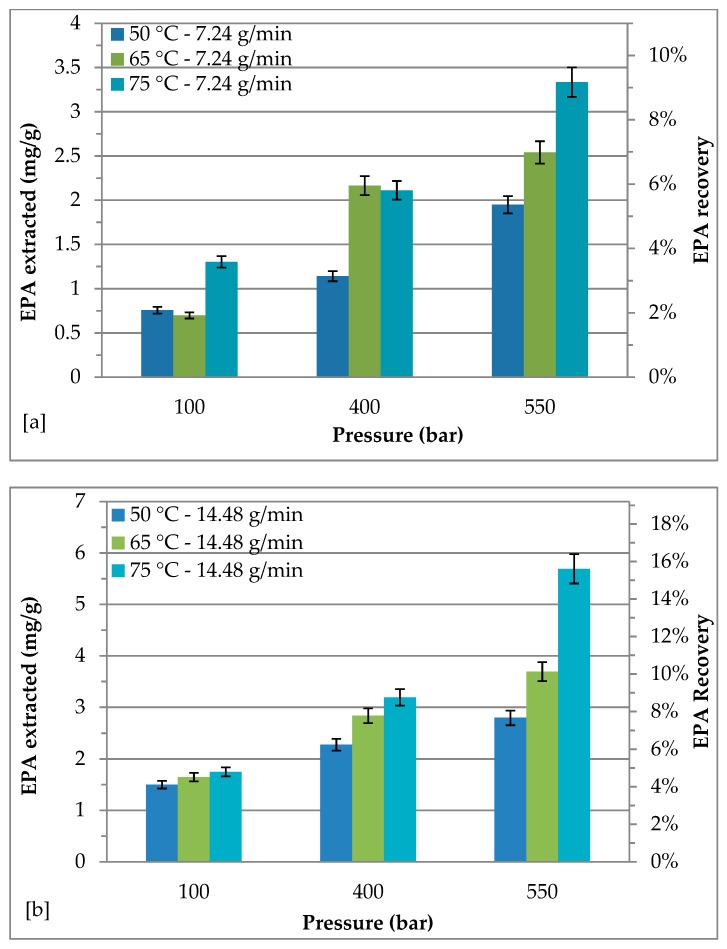
Effect of different pressure and temperature on recovery of eicosapentaenoic acid (EPA): (**a**) CO_2_ flow rate = 7.24 g/min; (**b**) CO_2_ flow rate = 14.48 g/min.

*Nannochloropsis* sp. has traditionally been studied as a source of ω-3 PUFAs, due to its high content in EPA. Indeed, as shown in Table 4, *Nannochloropsis* sp. lipids have a higher content of EPA (36.51 mg/g). As Figure 2 shows, the increase of pressure and the increase of temperature enhanced the EPA recovery at CO_2_ flow rates of 7.24 g/min and of 14.48 g/min. At CO_2_ flow rates of 7.24 g/min, the EPA recovery increased from 2.07% to 5.34% when the pressure grew from 100 bar to 550 bar at 50 °C. The same behavior was observed for extractions at 65 °C and at 75 °C. Gradually increasing extraction temperature from 50 °C to 75 °C, EPA recovery was gradually increased from 5.34% to 9.13%, excluding the EPA recovery at 65 °C and 100 bar. The maximum EPA recovery of 15.59% was achieved at 75 °C and 550 bar with a CO_2_ flow rate of 14.48 g/min, which was two folds higher in comparison with respect to EPA recovery at 50 °C and 550 bar. At 100 bar, EPA recovery was found to be almost the same at the three explored temperatures (55, 65 and 75 °C). 

Results also showed that by increasing CO_2_ flow rate from 7.24 to 14.48 g/min, EPA recovery grew up to 2.4 fold. 

The extraction by using supercritical CO_2_ is a complex process, during which different factors come into play, such as the gas distribution into the reactor and the molecular interactions between the solvent and the solute(s). According to several works reported in the literature [13,14,28,29,30], an optimum CO_2_ flow rate can be determined depending on the selected solute(s), above which the solvent manifests a negative influence on the extraction efficiency mainly due to a possible mass transfer resistance. Thus, in a supercritical fluid extraction, the best flow rate conditions should be determined by taking into account the temperature, the pressure, the geometry and the volume of the extractor, as well as the solute(s) nature. Recently, Di Sanzo et al. [16] evaluated the SF-CO_2_ extraction of astaxanthin, lutein, and FAs recoveries and purities at different pressures, temperatures and CO_2_ flow rates. The experimental data showed that the maximum recovery of astaxanthin and lutein could be achieved at low temperature and high pressure (50 °C and 550 bar, respectively) adopting the lowest investigated CO_2_ flow rate (3.62 g/min), while a higher flow rate (14.48 g/min) increased astaxanthin and lutein purities. Molino et al. [20] studied the effect of extraction pressure (250, 400 and 550 bars) and temperature (50 and 65 °C) at CO_2_ flow rates of 7.24 and 14.48 g/min for EPA recovery from *Nannochloropsis gaditana* biomass during SF-CO_2_ extraction, resulting in a maximum EPA recovery of 27.4% at 65 °C and 250 bars with a CO_2_ flow rate of 7.24 g/min. 

### 2.4. Effect of Different Pressure and Temperature on DHA Recovery with a CO_2_ Flow Rate of 7.24 and 14.48 g/min 

**Figure 3:**
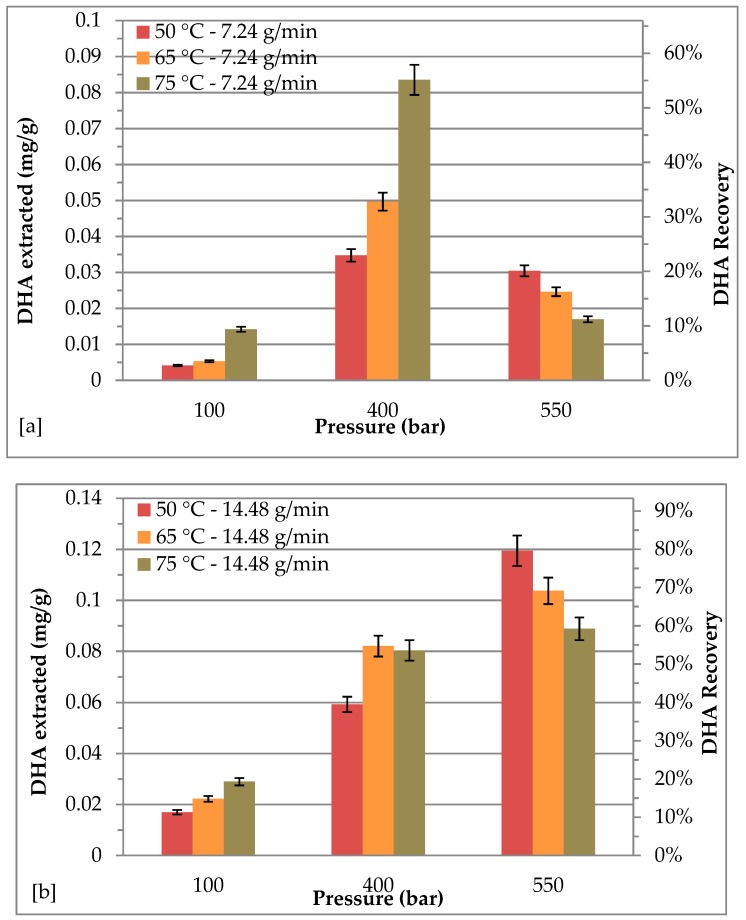
Effect of different pressure and temperature on recovery of DHA: (**a**) CO_2_ flow rate = 7.24 g/min; (**b**) CO_2_ flow rate = 14.48 g/min.

Figure 3 shows the effect of extraction pressure and temperature at CO_2_ flow rates of 7.24 and 14.48 g/min for DHA recovery. The results illustrate that a significant variation has been found in the DHA recovery (2.75–79.63%). The maximum recovery of DHA, equal to 79.63%, was achieved at 550 bars with a CO_2_ flow rate of 14.48 g/min and 50 °C. At CO_2_ flow rate of 7.24 g/min, DHA recovery increased from 100 bar to 400 bar and decreased from 400 bar to 500 bar; while at CO_2_ flow rate of 14.48 g/min, the higher the pressure, the higher the DHA recovery. For both the CO_2_ flow rates, at 100 bar and at 400 bar, the recovery increased with temperature; while at 550 bar, the higher the temperature, the lower the recovery. By using a CO_2_ flow rate of 14.48 g/min, DHA recovery enhanced from 11.32 to 79.63% by increasing extraction pressure from 100 bar to 550 bar at 50 °C, and a similar trend was observed at 65 °C and 75 °C with lower DHA recovery. These results clearly show that DHA recovery can be increased by a proper choice of the extraction conditions. In any case, DHA recovery was lower than 80%, therefore further efforts are needed to improve the DHA extraction process. Several studies report that higher temperatures may lead to thermal degradation of compounds, while higher pressures may reduce the diffusivity of the supercritical fluid into the matrix, resulting in a decrease of the extraction yield [6,11,32,33,34,35]. In addition, higher temperature and pressure could imply the waxes formation [36]. Di Sanzo et al. [16] found that higher extraction temperature and pressure allowed a decrease of recovery of astaxanthin and lutein, which could be possible, due to thermal degradation. At the same time, higher temperature could enhance the extraction yield, which leads to greater impurity content, as observed in the HPLC analysis profile [9,10]. Furthermore, several studies highlighted similar effects of pressure and temperature on the recovery of several bioactive compounds [22,35,37].

### 2.5. Effect of Different SF-CO_2_ Extraction Conditions on FAs Composition

Only a few FAs were significantly extracted among the 16 species investigated, such as heptadecanoic acid, palmitoleic acid and cis-5,8,11,14,17 eicosapentaenoic acid (Table 2). However, it is worth noting that they were the most abundant FAs in the lipid extracts (approx. 59–85% of total FAs), as it also was found by Lin et al. [38]. These FAs are normally treated as the main components for microalgal derived bioactive compounds, which have high nutrition value, and they are useful for human health in preventing several diseases [5,27,39,40,41,42,43,44]. Despite changes in FAs profile, it is conceived that the different extraction conditions could be more selective for specific FAs (extracted as triacylglycerols form). For example, CO_2_ flow rate of 14.48 g/min supports the recovery of cis-5,8,11,14,17 EPA at each fixed pressure and temperature. The maximum recovery (15.6%) of cis-5,8,11,14,17 EPA at 75 °C, 550 bar with a CO_2_ flow rate of 14.48 g/min was obtained, while similar recovery of heptadecanoic acid was achieved at 75 °C, 100 bar with a CO_2_ flow rate of 7.24 g/min.

This study suggests that it would be worth considering SF-CO_2_ extraction conditions for FAs profile analyses in microalgae.

### 2.6. Comparison with Literature Works

In Table 3, the comparison between the adopted operational conditions during SF-CO_2_ and those of similar literature works is reported. Table 3 also reports the main results in terms of lipid extraction yield and the ratio between the CO_2_ and the dried biomass. However, it does not immediately identify the optimal conditions for lipids extraction—primarily due to the differences in microalgae species, and, in addition, to the multiple variables of the entire extraction process (such as fixed pressure, temperature, extraction time and flow rate). It is possible to observe that the highest lipids extraction obtained in this work assume values, similar to those of Hernandez et al. [45].

## 3. Materials and Methods 

### 3.1. Microalgal Biomass and Chemical Composition 

Lyophilized biomass of *Nannochloropsis* sp. was collected from Archimede Ricerche srl, A&A F.lli Parodi Group, Italy, with a mesh particle size of about 10–35 μm. The biomass was stored at −20 °C in a vacuum plastic bag to avoid any biochemical composition variation and to keep it at room conditions before use. The chemical composition of lyophilized *Nannochloropsis* sp. in terms of humidity, ash, total dietary fiber (TDF), carbohydrates, proteins, FAs, saturated fatty acids (SFAs), monounsaturated fatty acids (MUFAs), PUFAs, specifically EPA and DHA reported in Table 4, was analyzed prior extraction tests by using standard methods described in our previous publications [20,49]. 

### 3.2. Chemicals 

Rivoira, Italy provided CO_2_ (99.999% purity) that was used for supercritical fluid extraction. The standards (FAs) that were used for the GC calibrations were of analytical grade and purchased from Sigma Aldrich, USA. All the used solvents were of HPLC grade and purchased from Sigma Aldrich, USA.

### 3.3. Mechanical Pre-Treatment of Biomass

In order to improve FAs recovery, the mechanical pre-treatment conditions were optimized in terms of biomass and diatomaceous earth mixing ratio, rotation speed, and pre-treatment time by means a Retsch PM200 planetary ball mill [13]. The jars of the mill were filled with 2 g of *Nannochloropsis* sp. biomass, that was mixed with diatomaceous earth (DE), with a mixing mass ratio of 0.5/1.0/2.0 DE/biomass w/w, respectively. 

For the pre-treatment, three sequential experiments were carried for different pre-treatment time 2.5/10 min by varying rpm (200/300/400/500/600 rpm). After the mechanical pre-treatment, FAs extraction was carried using an accelerated solvent extractor (ASE) with a chloroform:methanol:water (1:2:0.75 *v*/*v*) mixture as solvent at 50 °C and 100 bar, carrying out two consecutive extraction cycles each one of 10 min for total extraction time of 20 min. 

### 3.4. CO_2_ Supercritical Extraction Experiments

**Figure 4:**
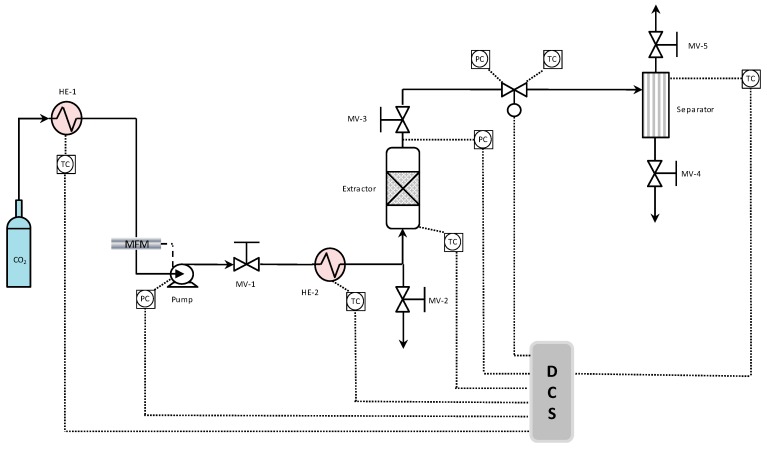
Bench scale SF-CO_2_ extraction unit schematization. **Note:** HE: Heat exchanger; MFM: Mass flow meter; MV: Manual valve; DCS: Distributed control system; PC: Pressure control; TC: Temperature control.

The SF-CO_2_ extraction method was carried out by using a bench scale extraction unit, as described in our previous studies [16,18]. The extraction unit had a heating capacity up to 250 °C and CO_2_ compression capacity up to 680 bar. The extraction unit can control the inlet and the outlet pressure with an accuracy of 0.6 mbar, and the CO_2_ flow rate was controlled by means of an LPN/S80 ALG 2.5, Sacofgas, Italy flow meter. The inlet flow rate was adjustable until 25 mL/min and controlled using the expanded gas. The temperature was monitored using thermocouples, where micrometric valves control the inlet and outlet flow streams. The cylindrical extraction vessel had a capacity of 50 mL (D = 1.35 cm, H = 35 cm), which was filled with pretreated biomass, including diatomaceous earth, and 44 g glass beads of 3 mm to increase the contact of carbon dioxide with microalgae, and, at the same time, to avoid the biomass caking. Moreover, at the bottom of the extraction vessel, metal frit filters were used with a pore diameter of 5 μm to avoid the biomass transfer. The extraction unit was equipped with acoustic and visual high-pressure alerts and, as a primary security system, a rupture disk was installed. All the parameters were controlled through a Distributed Control System (DCS). A schematization of bench scale extraction unit are reported in Figure 4. 

The effect of the variation of operative conditions, such as pressure (P) in the range 100–550 bar, CO_2_ flow rates 7.24 and 14.48 g/min, and temperature (T) in the range 50–75 °C on EPA and DHA extraction from *Nannochloropsis* sp. was investigated, while the biomass loading was kept constant (~2.0 g). The detailed experimental conditions are shown in Table 5. For each experimental test, five extraction cycles of 20 min each (extraction time = 20–100 min) were carried out. However, the results of the whole extraction process have been presented in this article.

The influence of the adopted operating conditions was studied on the recovery of total lipid, EPA and DHA. The amount of the extracted compounds from microalgae was expressed in terms of weight of the compound/weigh of dry biomass:

(1) $$\mathrm{Compound}\;\mathrm{extracted}\;(mg/g)=W_{C,i}/W_{M},$$

where W_C,i_ is the weight of the extracted EPA/DHA (mg); and, W_M_ is the weight of microalgae on a dry basis (g). Moreover, for each class, the recovery was compared with respect to the theoretical content.

The recovery percentage EPA/DHA was calculated from Equations (2).

(2) $$\mathrm{Recovery}\;\left(\%\right)=W_{C,i}/W_{T}\times 100,$$

where W_T_ is the theoretical weight of EPA/DHA (mg).

Furthermore, the purity percentage of lipid was also calculated, by using Equation (3)

(3) $$\mathrm{Purity}\;\left(\%\right)=W_{L}/W_{E}\times 100,$$

where W_L_ is the weight of total lipid extracted (mg), and W_E_ is the total weight of the extract (mg).

The percentage of FAs extracted was calculated by using Equation (4):

(4) $$\mathrm{FAs}\;\mathrm{Extracted}\;\left(\%\right)=SFA_{E}+MUFA_{E}+PUFA_{E}/\sum FAs_{T}\times 100,$$

where SFAE, MUFAE and PUFAE are the total amount of SFA extracted, the total amount of MUFA extracted, and the total amount of PUFA extracted, respectively (Table 1), and FAsT is the total theoretical content of FAs (Table 2).

Each experimental condition was investigated three times, and for each condition, the standard deviation (SD) value was calculated. After the SF-CO_2_ extraction, the extracts were stored in the dark at −80 °C before analyzing the total EPA contents using GC-FID.2.5. Analytical Methods 

After the mechanical pre-treatment, total lipids were analyzed by gravimetric analysis, and, more precisely, by following the Bligh and Dyer method [50]. Since a mixture of non-polar/low polarity organic solvent and polar organic solvent allows to extract a higher amount of lipids, a chloroform/methanol mixture (1:2 *v*/*v*) was used as extractant. 

FAs compositions were identified by GC separation of the corresponding methyl esters (FA methyl esters C8-C24). Lipids fraction was methylated through base-catalyzed transmethylation, according to BDS EN ISO 12966-2:2017 standard method [51]. More precisely, NaOH solution in methanol (0.5 M, 6 mL) and a spatula of boiling chips were added to a known quantity of extract (about 100 mg). The sample was transferred to a 50 mL one-mark volumetric Erlenmeyer flask that was connected to a reflux condenser to boil the sample for about 10 min. At the end of boiling, the apparatus was removed from the heat source, and 6 mL of n-hexane from the top of the condenser and followed by 7 mL of the BF3 catalyst in methanol (14%) (B1252 Aldrich) were added. The sample boiled again for 30 min, and 5 mL of isooctane were added at the end of the reaction. A 20 mL sample of a saturated NaCl solution was added and swirled, and the second aliquot of saturated NaCl solution was added until it filled up to the neck of the flask. The total upper layer (2−4 mL) was taken and then transferred to a GC glass vial. 

The chromatographic analysis was carried out using a 7820A GC-FID that was equipped with an HP-88 100 mt × 0.25 mm × 0.2 µm column (Agilent) for SFAs, MUFAs and PUFAs measurements. 

This chromatographic is composed of a high polarity bis (Cyanopropyl) siloxane stationary phase, and it was chosen for its high resolution of positional and geometric isomers of fatty acid methyl esters. According to the chromatographic conditions reported in the standard method UNI ISO 12966-4 [52], the injector, as well as the detector temperature, was maintained at 250 °C. The column was maintained at 120 °C for 5 min, followed by temperature ramping at 4 °C/min to 240 °C and held for a further 10 min at 240 °C. Nitrogen (purity ≥ 99.9999%) was used as carrier gas with a linear velocity of 30 cm/s (flow rate approx. 1.0 mL/min) and a split ratio of 1:100. The sample injection volume was 1 µL. The FAs characterization was carried out for each extraction condition, and an internal analytical standard of the tricosanoic acid (C:23) was used for the quantification of fatty acid methyl esters. A mixture of 37 fatty acid methyl esters (C4–C24) (Supelco FAME 37, CRM47885) was purchased from SIGMA-Aldrich (H5149), St Louis, MO, USA and was used for the quantitative analysis.

## 4. Conclusions

The increasing interest in exploiting microalgae as a source of commercial products with several health benefits has led to an ongoing search for high value-added compounds, e.g., EPA and DHA. In this study, the most influencing extractive conditions for lipid recovery from microalgae *Nannochloropsis* sp. biomass using SF-CO_2_ extraction were investigated. Results show that the maximum lipid extraction of 18.39 mg/g, with a purity of 19.51%, was obtained at 75 °C and 550 bar with a CO_2_ flow rate of 14.48 g/min. The highest EPA recovery (15.59%) was achieved at 550 bar with a CO_2_ flow rate of 14.48 g/min and 75 °C. The highest DHA recovery (79.63%) was obtained by working at 50 °C and 400 bar with a CO_2_ flow rate of 14.48 g/min, while it was assumed that higher temperatures could lead to the thermal degradation of DHA molecules. Experimental data indicated beneficial information to increase recovery of EPA and DHA from microalgal biomass. Results from this study allow the choice of the best SF-CO_2_ extraction conditions, to obtain the maximum recovery or the maximum purity of selected FAs.

## Figures and Tables

**Table 1:** Effect of supercritical fluid-carbon dioxide (SF-CO_2_) extraction conditions on extraction yield and lipid recovery from *Nannochloropsis* sp. biomass.

Experimental Code	Extraction Yield (mg/g)	Extracted Total Lipids (mg/g)	Cumulative Lipids Purity (%)	Extracted FAs (mg/g)	Extracted FAs (%)	SFAs (mg/g)	MUFAs (mg/g)	PUFAs (mg/g)
E_01	20.9	5.60	26.79	5.33	4.84	1.53	1.95	1.85
E_02	55.92	6.70	11.98	6.21	5.64	2.11	2.32	1.78
E_03	56.64	9.19	16.23	8.12	7.44	2.57	2.96	2.67
E_04	40.66	8.85	21.76	7.60	6.90	4.78	1.53	1.29
E_05	66.42	9.38	14.12	8.90	8.08	2.66	2.80	3.45
E_06	75.84	11.70	15.42	10.13	9.19	3.13	3.64	3.36
E_07	14.74	2.25	15.29	0.095	0.09	0.04	0.03	0.03
E_08	50.82	10.24	20.15	9.67	8.78	3.17	3.48	3.02
E_09	74.3	12.51	16.83	11.69	10.61	3.28	4.11	4.29
E_10	43.86	8.07	18.41	7.41	6.73	2.65	2.74	2.03
E_11	58.26	10.37	17.79	9.26	8.41	2.93	3.59	2.74
E_12	74.72	11.69	15.65	11.19	10.16	3.32	4.05	3.82
E_13	51.22	9.66	18.86	7.86	7.14	4.69	1.48	1.70
E_14	77.92	12.27	15.75	11.67	10.59	3.27	4.44	3.96
E_15	79.90	14.13	17.69	13.45	12.21	3.93	4.72	4.80
E_16	16.38	5.84	35.67	4.23	3.84	1.86	0.58	1.79
E_17	72.78	14.30	19.65	13.74	12.47	4.23	5.12	4.40
E_18	94.28	18.39	19.51	17.56	15.94	4.74	5.89	6.92

**Note:** Standard deviation was less than 5% in all operative conditions. Where: T: Temperature; P: Pressure; CO_2_ FR: CO_2_ flow rate.

**Table 2:** Composition of FAs extracted from *Nannochloropsis* sp. Biomass: Comparison between different SF-CO_2_ extraction conditions.

Experimental Code	FATTY ACIDS PROFILE – Recovey (%)														
	**OA**	**LA**	**TA**	**PA**	**PeA**	**HA**	**AA**	**PaA**	**EA**	**MY**	**NA**	**ETA**	**LiA**	**EPA**	**DHA**
**E_01**	38.40	3.20	4.45	9.39	0.00	8.57	5.86	4.35	29.55	3.76	9.84	0.00	18.51	2.07	0.00
**E_02**	48.36	0.57	2.06	2.66	0.34	13.47	7.10	6.57	29.35	5.72	4.01	0.10	10.12	3.13	23.17
**E_03**	55.61	0.56	1.73	3.46	1.09	16.09	9.64	8.11	24.69	7.48	7.48	0.12	11.57	5.34	20.47
**E_04**	6.42	0.00	2.70	0.00	29.46	30.60	0.00	4.15	99.32	0.37	0.00	2.78	6.14	1.91	3.54
**E_05**	34.19	2.99	1.44	39.13	0.28	16.45	9.14	8.01	22.82	8.05	4.41	7.72	10.45	5.93	33.13
**E_06**	5.30	0.48	2.20	3.32	0.37	21.89	5.89	9.83	27.42	9.47	9.86	0.11	13.26	6.96	30.93
**E_07**	66.20	0.00	0.00	97.58	0.14	4536	0.18	6.83	3.18	10.25	15.08	0.00	32.69	3.57	9.46
**E_08**	36.71	8.97	2.36	37.18	0.36	19.52	10.42	10.01	33.69	8.99	5.85	0.13	13.87	5.78	55.69
**E_09**	36.71	3.22	3.25	3.07	1.22	22.31	7.01	10.82	33.30	11.62	11.47	0.28	15.59	9.13	11.32
**E_10**	12.32	2.23	1.58	88.31	0.07	17.33	4.14	7.75	25.76	6.53	5.73	0.11	7.91	4.11	34.48
**E_11**	30.92	5.05	3.29	0.00	0.32	20.13	5.72	9.93	43.06	8.39	8.02	0.02	6.85	6.24	39.49
**E_12**	45.94	1.06	2.59	1.98	0.47	23.02	6.84	11.15	38.62	10.47	9.34	9.00	3.18	7.66	79.63
**E_13**	3.02	0.00	2.80	0.00	0.00	12.23	80.59	5.62	1.00	0.23	0.23	0.00	0.44	4.51	14.82
**E_14**	72.03	2.59	2.51	1.99	0.64	22.08	7.01	11.09	31.80	10.55	17.59	2.35	14.43	7.77	54.71
**E_15**	53.68	0.75	2.92	5.06	0.63	25.38	14.50	12.49	45.52	12.89	12.65	0.00	16.91	10.12	69.15
**E_16**	7.59	0.00	1.50	0.00	0.00	5.31	30.10	1.94	3.22	0.30	0.90	0.00	0.28	4.79	19.31
**E_17**	76.91	0.87	2.97	0.76	1.45	27.96	12.25	14.01	39.64	12.60	13.50	0.10	18.93	8.74	53.55
**E_18**	64.15	6.42	4.24	5.15	2.00	31.71	11.34	14.96	49.08	17.94	17.81	0.00	19.38	15.59	59.22

**Note:** Standard deviation was less than 5% in all operative conditions; Where: T: Temperature; P: Pressure; CO_2_ FR: CO_2_ flow rate; OA: Octanoic acid; LA: Lauric acid; TA: Tridecanoic acid; PA: Palmitic acid, PeA: Pentadecanoic acid; HA: Heptadecanoic acid; AA: Arachidic acid; PaA: Palmitoleic acid: EA: Elaidic acid; MA: Myristoleic acid; NA: Nervonic acid; LiA: Linoelaidic acid; DHA: Docosahexaenoic acid; ETA: cis-5,8,11,14 Eicosatetraenoic acid; EPA: cis-5,8,11,14,17 Eicosapentaenoic acid.

**Table 3:** Comparison between the operational conditions, adopted for SF-CO_2,_ the main results of this work, and other examples from the literature.

Microalgae Specie	SF-CO_2_ Operational Conditions				Lipid Extraction Yield	CO_2_/Dried Biomass	Ref.
	Pressure	Temperature	Extraction Time	Flow Rate			
	bar	°C	min	g/min	%	g/g	
*Pavlova* sp.	306	60	360	--	17.90	--	[26]
*Hypnea charoides*	379	40	120	1.40	58.00	30	[28]
*Isochrysis T-ISO*	300	45	90	6.70	7.70	1200	[45]
*Nannochloropsis gaditana*	300	45	90	6.70	7.90	1200	
*Tetraselmis* sp.	300	45	90	6.70	11.10	1200	
*Scenedesmus almeriensis*	300	45	90	6.70	10.10	1200	
*Nannochloropsis oculata*	250	50	240	25.00	15.00	214	[46]
*Chlorella*	350	50	--	--	50.00	30	[47]
*Schizochytrium* sp.	276	50	360	353.53	10.00	636	[48]
*Spirulina* sp.	276	50	360	353.53	9.00	636	
*Nannochloropsis* sp.	550	75	100	7.24	8.22	362	This work
	550	75	100	14.48	12.08	724	

**Table 4:** Chemical composition of *Nannochloropsis* sp. biomass.

Chemical Composition of *Nannochloropsis* sp.	Content (mg/g)
Humidity (mg/g wet)	19.03
Total dietary fiber (TDF)	196.90
Carbohydrates	350.87
Proteins	267.04
Lipids	153.22
Ash	83.17
**Fatty acid composition**	
Octanoic acid	0.17
Lauric acid	1.10
Tridecanoic acid	2.33
Palmitic acid	0.26
Pentadecanoic acid	2.95
Heptadecanoic acid	12.53
Arachidic acid (o arachico)	3.83
**Σ SFAs**	**23.17**
Palmitoleic acid	25.88
Elaidic acid	0.45
Myristoleic acid	4.84
Nervonic acid	5.22
**Σ MUFAs**	**36.39**
cis-8,11,14-Eicosatrienoic acid (ω-6)	8.03
Linoelaidic acid (ω-6)	5.91
cis-5,8,11,14,17-Eicosapentaenoic acid (ω-3)-EPA	36.51
cis-4,7,10,13,16,19-Docosahexaenoic acid (ω-3)-DHA	0.15
**Σ PUFAs**	**50.60**

**Note:** Standard deviation was less than 5% in all operative conditions.

**Table 5:** The experimental condition during SF-CO_2_ extraction for lipid recovery from *Nannochloropsis* sp. biomass.

Experiment Code	Temperature (°C)	Pressure (bar)	CO_2_-Flow Rate (g/min)
E_01	50	100	7.24
E_02	50	400	7.24
E_03	50	550	7.24
E_04	65	100	7.24
E_05	65	400	7.24
E_06	65	550	7.24
E_07	75	100	7.24
E_08	75	400	7.24
E_09	75	550	7.24
E_10	50	100	14.48
E_11	50	400	14.48
E_12	50	550	14.48
E_13	65	100	14.48
E_14	65	400	14.48
E_15	65	550	14.48
E_16	75	100	14.48
E_17	75	400	14.48
E_18	75	550	14.48

**Note:** Biomass loading was expressed on dry basis.
